# Influence of access cavity design on the structural integrity of endodontically treated permanent mandibular 1^st^ molar teeth: a quasi-experimental study

**DOI:** 10.3389/fdmed.2026.1837954

**Published:** 2026-06-15

**Authors:** Jayesmin Akter, A. K. M. Bashar, Mohammad Tofazzel Hossain, Mohammad Mahmudul Hasan Khan, Madhab Paul, Md Ali Asgar Moral, Mahmud Mohammed, Abedelmalek Kalefh Tabnjh, Apitchaya Pruksametanan, Siddharthan Selvaraj

**Affiliations:** 1 Conservative Dentistry & Endodontics Dental Unit, TMSS Medical College, Bogura, Bangladesh; 2 Department of Conservative Dentistry and Endodontics, Bangladesh Medical University, Dhaka, Bangladesh; 3 Department of Conservative Dentistry and Endodontics, Shaheed Suhrawardy Medical College, Dhaka, Bangladesh; 4 Department of Conservative Dentistry and Endodontics, Sir Salimullah Medical College, Dhaka, Bangladesh; 5 College of Medicine and Dentistry, Cairns Campus, James Cook University, Cairns, QLD, Australia; 6 Department of Cariology, Institute of Odontology, The Sahlgrenska Academy, University of Gothenburg, Gothenburg, Sweden; 7 Department of Applied Dental Sciences, Faculty of Applied Medical Sciences, Jordan University of Science and Technology, Irbid, Jordan; 8 Department of Pathology, Saveetha Medical College and Hospital, Saveetha Institute of Medical and Technical Sciences, Chennai, Tamil Nadu, India; 9 Institute of Dentistry, Suranaree University of Technology, Nakhon Ratchasima, Thailand; 10 Faculty of Dentistry, University of Puthisastra, Phnom Penh, Cambodia; 11 Norxin International Science and Technology Cooperation Base, Xi'an City, Shaanxi Province, China

**Keywords:** access cavity, conservative, conservative access cavity design, endodontically treated teeth, fracture resistance

## Abstract

**Background:**

Endodontically treated teeth are increasingly prone to fracture over time, and their resistance to fracture is closely related to the amount of coronal tooth structure removed during access cavity preparation. Preservation of sound tooth structure through appropriate access cavity design is therefore essential for maintaining long-term tooth strength and clinical performance. Accordingly, this study evaluated the influence of traditional and conservative access cavity designs on the fracture resistance of endodontically treated mandibular first molars.

**Methods:**

Forty recently obtained human permanent mandibular first molars were arbitrarily allocated into four groups, each comprising ten teeth: traditional access cavity (TradAC), contracted access cavity (ConsAC), truss access cavity (TrussAC), and intact teeth (control). The experimental groups underwent root canal treatment, and the access cavities were subsequently restored with composite resin to standardize coronal reinforcement prior to fracture testing. Fracture resistance was evaluated using a universal testing machine, and fracture patterns were analyzed under a stereomicroscope for fracture type classification. Statistical comparisons among groups were accomplished using one-way ANOVA, tracked by Tukey *post hoc* tests at *p* < 0.05.

**Results:**

Through this experiment, teeth prepared with TradAC exhibited significantly lesser fracture resistance compared with TrussAC and intact teeth (*p* < 0.05), while no substantial differences were observed between TrussAC and ConsAC or between TradAC and ConsAC (*p* > 0.05). Unrestorable fractures were more common in the TradAC, ConsAC, and TrussAC groups compared with the control group, although this difference was not statistically significant (*p* > 0.05). Based on these findings, truss access cavity design demonstrated higher fracture resistance than traditional access cavity design in endodontically treated teeth, and unrestorable fractures were more common than restorable fractures in teeth with access cavity preparations.

**Conclusion:**

These findings highlight that adopting conservative access cavity designs, particularly truss access cavities, may improve the structural durability of endodontically treated teeth and support more predictable long-term clinical outcomes.

## Introduction

Access cavity design is a critical determinant of the success and longevity of endodontic treatment. Endodontically treated teeth exhibit a higher susceptibility to fracture than vital teeth, primarily due to the loss of tooth structure during access preparation and canal instrumentation. Preservation of sound coronal dentin during endodontic procedures is therefore essential to maintain tooth strength and boost fracture resistance of fracture over time (1, 2). The extent of coronal tooth structure removed during access cavity preparation plays a crucial role in determining the fracture resistance of endodontically treated teeth (3–6).

Several studies have demonstrated that excessive removal of coronal and pericervical dentin during access cavity preparation adversely affects the mechanical behavior of teeth. Extensive access preparation reduces tooth stiffness, increases cuspal deflection, and compromises resistance to functional occlusal loads, thereby predisposing teeth to catastrophic fractures (7–10). Consequently, fracture susceptibility has been shown to increase in direct proportion to the amount of tooth structure removed during endodontic treatment (11). Excessive removal of dentin necessary for access cavity formulation may therefore impair the tooth's ability to withstand functional occlusal loads (1, 2).

Traditional access cavity design entails the complete removal of the pulp chamber roof, with smoothly divergent axial walls, to attain straight-line access to the root canal orifices and facilitate their visualisation within the cavity outline. While this approach improves accessibility, it often results in a substantial loss of coronal dentin. In contrast, conservative access cavity preparation is based on the principle of “prevention of extension,” in which only the minimum tooth structure necessary to locate and instrument the canals is removed. This approach aspirations to preserve portions of the pulp chamber roof and pericervical dentin, which play vital tasks in distributing functional stresses and enhancing fracture resistance (12, 13). However, there are no standardized guidelines for conservative access cavity preparation, and the design largely depends on the operator's ability to balance dentin preservation with adequate canal localization.

More recently, truss access cavity design has been proposed as an extension of minimally invasive endodontics. This approach involves creating separate, small access openings aligned directly over individual root canal orifices while maintaining a dentinal bridge between them in multi-rooted teeth (14, 15). Cleaning, shaping, and obturation are performed through these minimal access openings, which are positioned directly over the canal orifices. As the lateral pulp horns and a significant portion of the pulp chamber roof remain intact, this design is expected to enhance fracture resistance by preserving critical coronal and pericervical dentin (16). In view of the growing emphasis on minimally invasive endodontics and limited comparative evidence, this study evaluated the fracture resistance of mandibular molars prepared with traditional, contracted, and truss access cavity designs (16). By preserving the pulp chamber roof and lateral pulp horns, truss access cavities are expected to boost fracture resistance by maintaining coronal tooth structure.

Although conservative and truss access cavity designs have been introduced to preserve tooth structure during endodontic treatment, the available evidence regarding their effect on fracture resistance remains limited and inconsistent. Furthermore, few studies have specifically compared traditional, contracted, and truss access cavity designs in permanent mandibular first molars. Therefore, this study was conducted to evaluate whether minimally invasive access cavity designs can improve structural preservation and fracture resistance while still allowing adequate endodontic access and treatment.

The purpose of using molars in this research study was to evaluate the Dental caries susceptibility, as well as the potential for Endodontic treatment, related to the location of these teeth within the oral cavity and their role in the mastication process. The first molars also have a high degree of occlusal loading because of their position as the only permanent molars, which increases the likelihood of developing a cusp fracture after receiving Endodontic treatment. In addition, the first molars have a more complicated anatomical structure than other permanent teeth, making them ideal candidates for further evaluating access cavity configurations and their fracture resistance.

The three access cavity designs were selected to compare the biomechanical effects of varying degrees of tooth structure preservation. Traditional access cavity (TradAC) represents the conventional approach that provides straight-line access and enhanced instrumentation visibility. Conservative access cavity (ConsAC) was included because it aims to preserve pericervical dentin and reinforce tooth strength while maintaining adequate canal accessibility. Truss access cavity (TrussAC) was chosen as an ultraconservative design intended to maximize preservation of dentinal structure between canal orifices. Comparing these three designs allows assessment of the relationship between access cavity extent and fracture resistance of endodontically treated teeth.

In view of these considerations, the overall objective of this research was to examine the impact of various access cavity designs on the fracture resistance of endodontically treated teeth. The specific objectives were as follows:

- To evaluate and compare the fracture resistance and fracture patter of endodontically treated mandibular permanent first molars prepared with traditional, contracted, and truss access cavity designs, and to compare these findings with intact teeth.
- To evaluate and compare the fracture patterns (restorable and unrestorable) associated with traditional, contracted, and truss access cavity designs.

This article is organized into the following sections: Section 2 explains the materials and methods used to prepare the access cavities, perform endodontic treatment, and evaluate fracture resistance. The experimental results and statistical analyses are presented in Section 3, and the findings are discussed in Section 4 with reference to existing literature and clinical implications. Finally, Section 5 summarizes the findings with future research directions.

## Materials and methods

This quasi-experimental study was conducted jointly at the Department of Conservative Dentistry and Endodontics, Bangabandhu Sheikh Mujib Medical University, and the Pilot Plant and Process Development Centre of the Bangladesh Council of Scientific and Industrial Research. Ethical approval was obtained from the Institutional Review Board of Bangabandhu Sheikh Mujib Medical University (BSMMU/2022/2249). Forty recently obtained human mandibular permanent first molars that endured the inclusion criteria were selected for the study. The teeth were collected from the patients who visited the Oral and Maxillofacial Department of Bangladesh Medical University. Teeth with patient age 20–60 years old, fully formed apices, absence of caries, restorations, cracks, root resorption, or previous endodontic treatment were included. Teeth with calcified canals, open apices, fractures, developmental anomalies, or resorption defects were excluded. Teeth were extracted for periodontal, orthodontic, or prosthodontic reasons and all extractions were performed by qualified oral and maxillofacial surgeons under standard clinical procedures. All soft tissue remnants and debris were removed using an ultrasonic scaler. Each tooth was examined under 2.5X magnification using dental surgical loupes to exclude pre-existing crown or root fractures, cracks, and craze lines. The buccolingual and mesiodistal dimensions were measured at the cervical margin using a digital calliper. To shrink the effect of variations in tooth size and morphology on the results, a homogeneous sample was established by selecting teeth with buccolingual dimensions of 9–11 mm and mesiodistal dimensions of 10–12 mm, as described in Table 1.

**Table 1:** Buccolingual (BL) and mesiodistal (MD) diameters in mm for n samples.

Group	*n*	BL	MD
TradAC	10	9.371 ± 0.262	10.616 ± 0.290
ConsAC	10	9.357 ± 0.237	10.531 ± 0.274
TrussAC	10	9.435 ± 0.185	10.452 ± 0.209
Control	10	9.556 ± 0.345	10.518 ± 0.364
*p*-value		0.330	0.657

**Figure 1:**
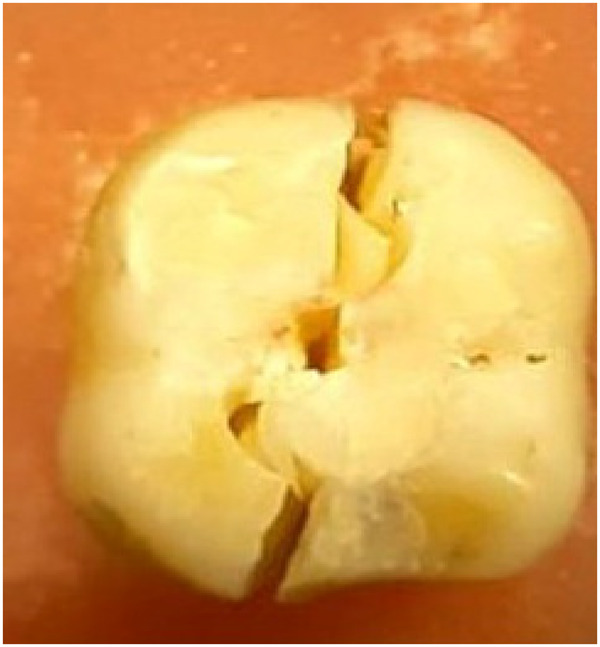
Unrestorable fracture.

**Figure 2:**
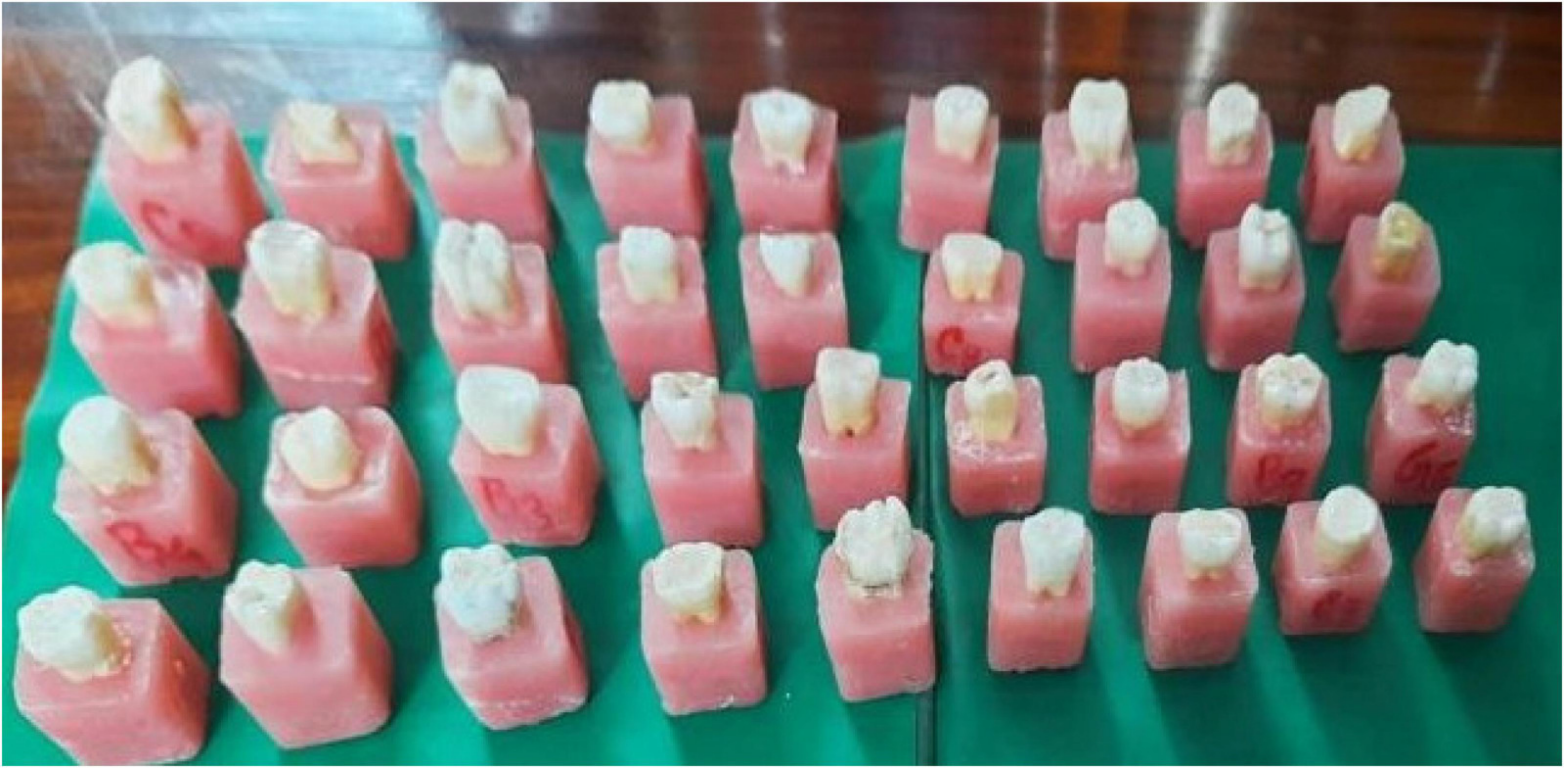
Teeth embedded in acrylic block.

**Figure 3:**
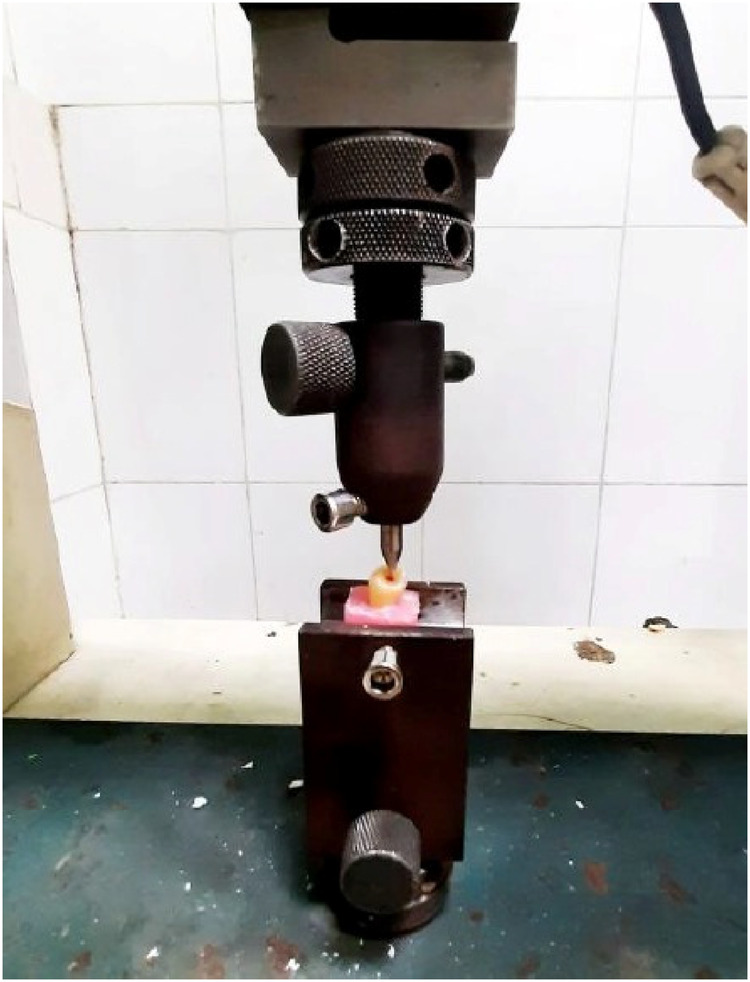
Fracture resistance test.

Preoperative radiographs of all extracted teeth were obtained to assess tooth morphology, root canal configuration, presence of open apices, calcifications, additional canals, and any pre-existing fractures. Figure 1 illustrates unrestorable fracture, Figure 2 displays the teeth embedded in acrylic block and Figure 3 shows the fracture resistance test.

The teeth were stored in normal saline until further procedures to prevent dehydration. Subsequently, the specimens were arbitrarily assigned into four groups (*n* = 10 per group) as follows: Group I-traditional access cavity design (TradAC); Group II-contracted access cavity design (ConsAC); Group III-truss access cavity design (TrussAC); and Group IV-intact teeth, which served as the control group.

**Figure 4:**
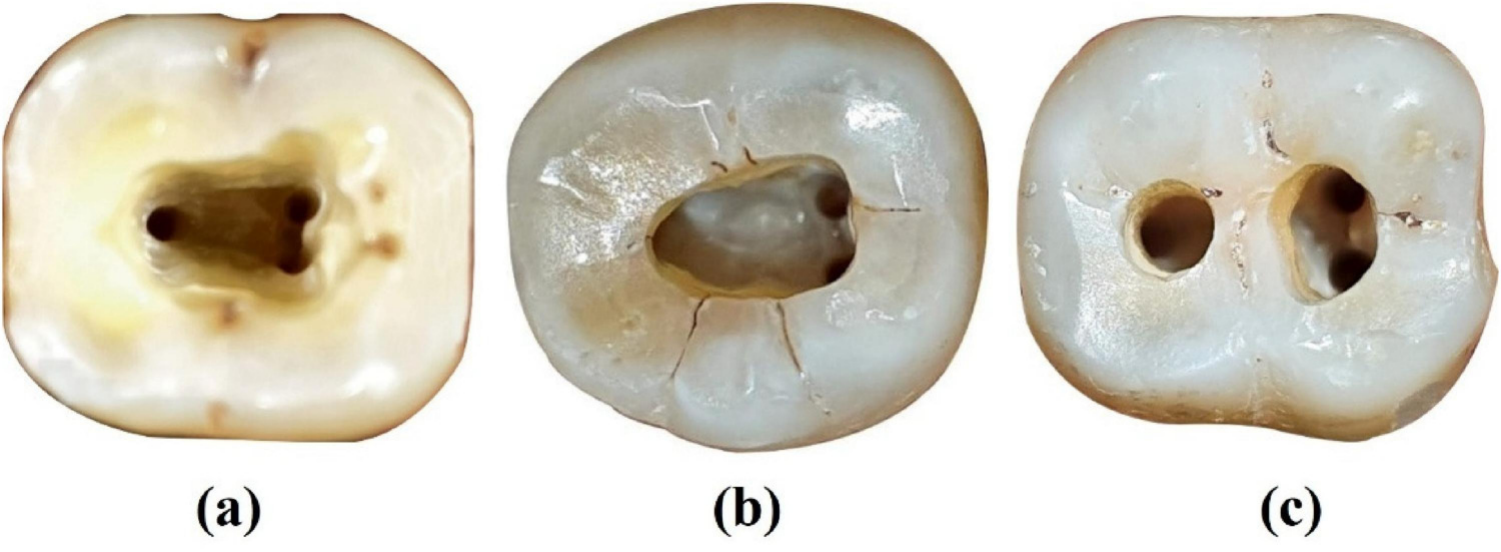
Access cavity design for each group: **(a)** traditional access cavity; **(b)** conservative access cavity; **(c)** truss access cavity.

Traditional access cavities were made using an Endo Access bur (Dentsply Maillefer, USA) in a high-speed handpiece with water cooling, in accordance with established guidelines. TradAC was arranged according to previously reported principles (17, 18), as shown in Figure 4A. ConsAC preparation was accomplished, ascending to the mesial quarter of the central fossa, with cavities covering the apical and distal aspects while retaining part of the chamber roof. Removal of mesiodistal, buccolingual, and circumferential pericervical dentin was minimized to preserve a portion of the pulp chamber roof while allowing identification of all root canal orifices (19). Initial access was directed toward the largest canal, usually the distal canal of the mandibular molar, using endodontic access burs. From the given canal orifice, the remaining canal orifices were searched, as displayed in Figure 4B. TrussAC was functioned by tipping part of the pulp chamber roof through carefully opened access. Coronal access was obtained immediately above the mesial pulpal horn. Access to the pulp chamber was obtained from the occlusal surface to the roof of the pulp chamber by orienting the bur parallel to the tooth's long axis and using a small endo-access bur in an oval buccolingual shape. The mesio-buccal and mesio-lingual orifices were confirmed using a DG16 probe. Subsequently, access to the pulp chamber was achieved by positioning the bur over the distal pulpal horn, and the distal orifices were verified with a DG16 probe. De-roofing above the canal orifices was done using a safe-ended diamond Endo Z bur (Dentsply, Maillefer, Switzerland) (14), leaving a truss in between the mesial and distal cavities. This conservative access approach retained the pulp chamber roof and lateral pulp horns by limiting the size of the access openings (15, 16), as illustrated in Figure 4C.

### Endodontic treatment

Following access cavity preparation, apical patency was designed using a size 10 K-file (Dentsply Maillefer, Ballaigues, Switzerland) beyond the apical foramen in the presence of a viscous chelating agent (Glyde; Dentsply Maillefer, Switzerland). Coronal flaring was performed using a ProTaper SX file (Dentsply Maillefer, Switzerland). Glide path preparation was initially achieved with a size 10 K-file and subsequently enlarged to a size 15 K-file. Working length was settled using a size 15 K-file by subtracting 1 mm from the length at which the file tip was detectable at the apical foramen, under 2.5X magnification.

Root canal instrumentation was completed using the ProTaper Gold rotary file system up to size F3, corresponding to an apical size of 0.30 mm. During instrumentation, canals were irrigated with 2 mL of 5.25% sodium hypochlorite using a 30-gauge needle, and apical patency was maintained by recapitulation with a size 15 K-file. After instrumentation, the smear layer was removed by irrigating with 2 mL of 17% ethylene-di-amine-tetra-acetic acid (EDTA), followed by a final rinse with 2 mL of 5.25% sodium hypochlorite. The canals were then dried with sterile paper points. Obturation was performed using a single-cone technique with gutta-percha cones (Dentsply Maillefer, Switzerland) and Sealapex sealer (Kerr, SybronEndo, Italy). Excess gutta-percha was removed from the canal orifices using a heated instrument, and the access cavities were finally cleaned with ethyl alcohol (12, 20).

### Teeth restoration

Upon completion of endodontic therapy, the access cavities were cleaned and etched using 37% phosphoric acid for 30 s, followed by thorough rinsing and gentle air-drying. A light-cured bonding agent (BeautiBond; Shofu Inc., Kyoto, Japan) was applied, air-thinned, and polymerized using a light-emitting diode curing unit 800 mW/cm^2^ for 30s. Packable composite restorations were placed in increments (3M ESPE, St. Paul, MN, USA), followed by finishing and polishing with a composite polishing system (Shofu, USA).

## Testing and analysis

This section outlines the fracture resistance testing protocol and summarizes the results and statistical comparisons among the four study groups. The impact of access cavity design on fracture resistance and fracture patterns is evaluated and discussed in this section.

### Fracture resistance test

All specimens were embedded vertically in self-cure acrylic resin blocks, with the roots exposed up to 2 mm below the cemento-enamel junction to allow assessment of fracture patterns. Fracture resistance testing was performed using a universal testing machine (Hounsfield, H10KS, UK). A compressive load was applied to the central fossa of each tooth using a stainless-steel rod with a rounded tip (2 mm diameter) at a crosshead speed of 0.5 mm/min, parallel to the long axis of the tooth. Loading was continued until fracture occurred, and the maximum fracture load was recorded in Newtons. Following fracture testing, specimens were researched under a stereomicroscope (Zeiss Stemi 508, Germany) at 2.5X enlargement to evaluate fracture patterns. Fracture patterns were categorized according to their position relative to the acrylic resin level, with fractures above considered restorable and those below considered unrestorable (21).

### Results and discussion

According to Table 2, the control group exhibited the highest fracture resistance, whereas the truss and contracted access cavity groups showed lower values, and the traditional access cavity group showed the lowest resistance. Statistically significant differences were observed between Group I (TradAC) and Group III (TrussAC), Group I (TradAC) and Group IV (control), Group II (ConsAC) and Group IV (control), and Group III (TrussAC) and Group IV (control) (*p* < 0.05). In contrast, no considerable alterations were found between Group I (TradAC) and Group II (ConsAC) or between Group II (ConsAC) and Group III (TrussAC) (*p* > 0.05), as mentioned in Table 3.

**Table 2:** Comparison of mean fracture resistance among groups (*n* = 40).

Groups name	Sample size (*n*)	Vertical load of fracture		*p*-value
		Mean ± SD		
Group I (TradAC)	10	687.4	± 207.6	< 0.001*
Group II (ConsAC)	10	791.5	± 251.4	
Group III (TrussAC)	10	1056.3	± 197.6	
Group IV (Control)	10	1403.6	± 264.2	

*Indicates a statistically significant difference (*p* < 0.05).

**Table 3:** Multiple pairwise comparison among groups.

Groups	Mean difference	Standard error	*p*-value
Group I vs. Group II	−104.08	103.72	1.000
Group I vs. Group III	−368.91	103.72	0.006*
Group I vs. Group IV	716.20	103.72	< 0.001*
Group II vs. Group III	−264.83	103.72	0.090
Group II vs. Group IV	612.12	103.72	< 0.001*
Group III vs. Group IV	347.29	103.72	0.011*

*Indicates a statistically significant difference (*p* < 0.05).

**Figure 5:**
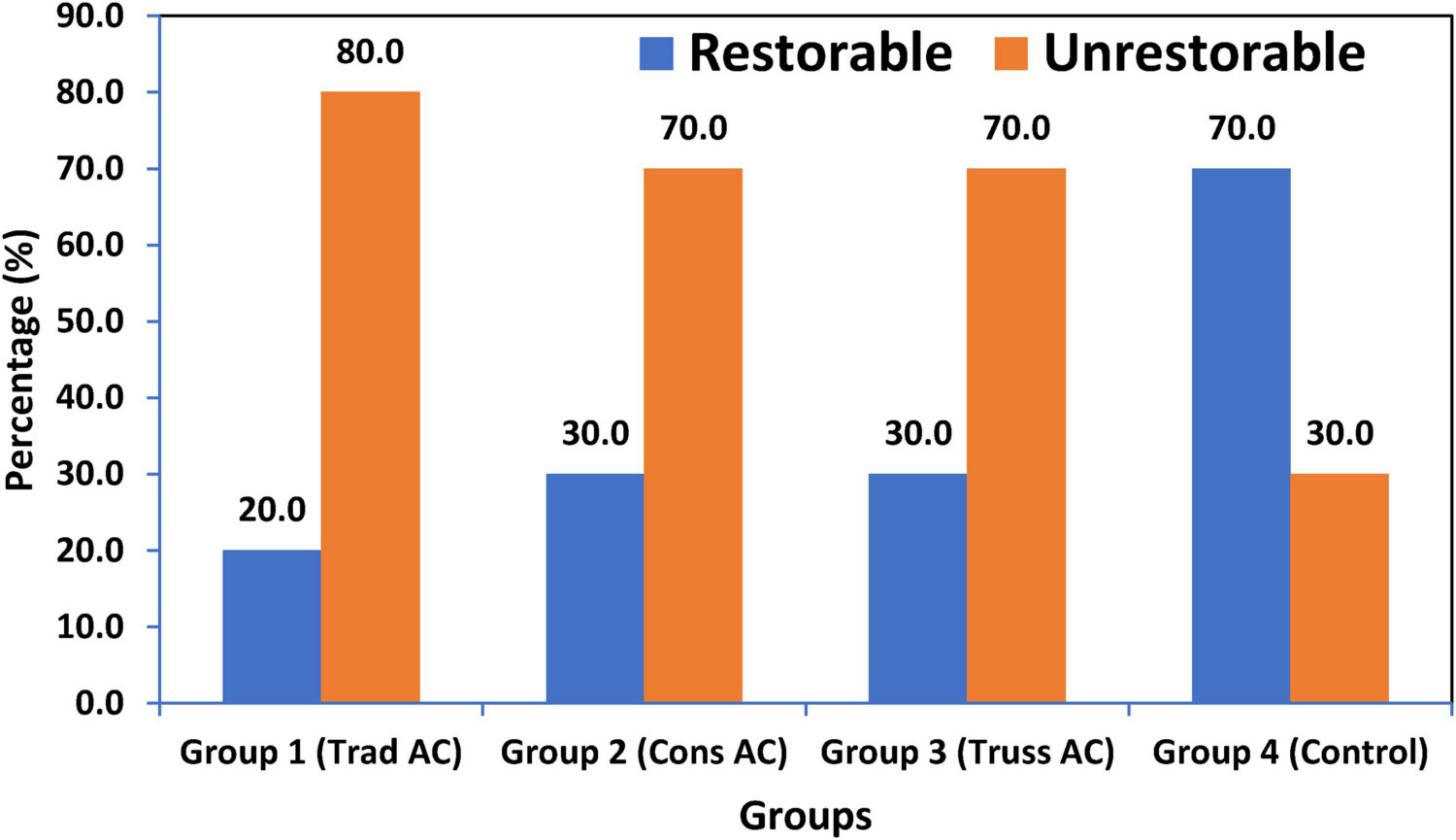
Fracture patterns in four group.

With respect to fracture patterns, unrestorable fractures were more common than restorable fractures in all experimental groups. In the control group, a higher proportion of restorable fractures was observed. However, the variations in fracture patterns among the study groups were not statistically noteworthy (*p* = 0.098), as explained in Figure 5.

The traditional access cavity group exhibited the lowest fracture strength compared with the contracted and truss access cavity groups, with the difference being statistically significant (*p* < 0.001). The mandibular molar tooth, the centre area of the tooth (i.e., occlusal enamel and dentin), tends to bear the high chewing forces (12). Because the bulk of the tooth structure, including the centre of the occlusal surface, must be removed during TradAC preparation, the tooth loses its natural capacity to withstand masticatory loads. Previous reports have demonstrated similar fracture resistance outcomes for traditional and truss access cavity groups (16, 22, 23). Contrasting findings across research may be due to differences in the types of teeth used, the restorative materials employed, and the methodologies employed.

Besides, the fracture resistance of teeth with TradAC and ConsAC did not differ notably (*P* = 1.000), consistent with a previous study. Based on the study by Özyürek (12), teeth with 3 residual walls were used, and the study was conducted on the TradAC and ConsAC groups. Although another study found a significant difference in fracture resistance between teeth with TradAC and ConsAC (24), the contradictory result may be attributed to the methodology employed in (25).

In this investigation, no major statistical difference in fracture resistance was observed between teeth prepared with contracted and truss access cavity designs (*p* = 0.090). Comparable findings have been reported in earlier studies evaluating truss access cavities (3, 16). A plausible explanation, as suggested in previous reports, is that both contracted and truss access cavity designs rely on very limited coronal openings, which may restrict canal visualization and instrumentation. Such limitations can compromise thorough debridement and, consequently, increase the restored tooth's susceptibility to fracture.

Beyond its effect on fracture resistance, reduced access cavity preparation may compromise endodontic efficiency by hindering canal identification and thorough debridement of pulp tissue and debris (15, 26). The collected data were analyzed with SPSS software (version 26; IBM Corp., Armonk, NY, USA). Group comparisons were analyzed using one-way ANOVA with Tukey–Kramer *post hoc* analysis, with statistical significance reported as *p*-values. Based on the results of the data analysis, there was an almost normal distribution of scores and fairly equivalent levels of variability among each group. Therefore, it can be concluded that all assumptions required to conduct the statistical analysis were met correctly. Besides, pairwise comparisons of fracture resistance were made between groups. When the fracture resistance of teeth in the TradAC, ConsAC, and TrussAC groups was compared with that of the control (intact teeth) group, a statistically significant deviation was observed in most studies (3, 12, 16). It may be due to intact teeth, with no loss of tooth structure. However, study by Plotino and his colleagues in the year 2017 (21), found no major distinction among the intact teeth, ConsAC, and TrussAC groups, owing to the minimally invasive access cavity design in both ConsAC and TrussAC.

Analysis of fracture patterns revealed that unrestorable fractures predominated over restorable fractures in the traditional, contracted, and truss access cavity groups, although this difference did not reach statistical significance (*p* = 0.098). Similar trends have been reported in earlier studies (3, 21). In contrast, intact teeth exhibited a greater proportion of restorable fractures. Extensive preparation associated with traditional access cavity design significantly reduces the amount of sound dentin, increasing tooth deformability and predisposing the tooth to unfavorable, non-restorable fractures. Even with improved preservation of pericervical dentin and the pulp chamber roof, contracted and truss access cavity designs may not fully prevent a reduction in fracture strength resulting from loss of tooth structure.

## Conclusion

Access cavity design impacts the fracture resistance of endodontically treated permanent mandibular molars; Truss access cavities provided greater fracture resistance than both traditional and contracted access cavities; however, fracture resistance of all three compared favorably. In all three groups, both types of fracture were found to occur more frequently than restorative type fractures.

This study was conducted under *in vitro* conditions with static axial loading, which does not fully replicate the complex oral environment, including periodontal ligament support, cyclic masticatory forces, and thermal variations. In addition, the sample size was limited as the sample size was selected based on previously published *in vitro* studies with similar methodologies, and only mandibular permanent first molars restored with composite resin were evaluated.

Further studies incorporating larger sample sizes, different tooth types, dynamic fatigue loading, thermocycling, and various restorative approaches are recommended to better simulate clinical conditions. Long-term clinical studies are also necessary to validate the fracture resistance benefits of conservative access cavity designs, particularly truss access cavities, in routine endodontic practice.

## Informed consent

The study participants provided written informed consent to participate in this study.

## Data Availability

The raw data supporting the conclusions of this article will be made available by the authors, without undue reservation.
